# Transcriptome Analysis Reveals the Effect of Stocking Density on Energy Metabolism in the Gills of *Cherax quadricarinatus* under Rice-Crayfish Co-Culture

**DOI:** 10.3390/ijms241411345

**Published:** 2023-07-12

**Authors:** Rui Jia, Yin Dong, Yiran Hou, Wenrong Feng, Bing Li, Jian Zhu

**Affiliations:** 1 Wuxi Fisheries College, Nanjing Agricultural University, Wuxi 214081, China; jiar@ffrc.cn (R.J.); 2020113024@stu.njau.edu.cn (Y.D.); houyr@ffrc.cn (Y.H.); 2 Key Laboratory of Integrated Rice-Fish Farming Ecology, Ministry of Agriculture and Rural Affairs, Freshwater Fisheries Research Center, Chinese Academy of Fishery Sciences, Wuxi 214082, China; fengwenrong@ffrc.cn

**Keywords:** ATP metabolic process, oxidative phosphorylation, oxidative stress, lipid metabolism, *Cherax quadricarinatus*

## Abstract

Stocking density is a crucial factor affecting productivity in aquaculture, and high stocking density is a stressor for aquatic animals. In this study, we aimed to investigate the effects of stocking densities on oxidative stress and energy metabolism in the gills of *Cherax quadricarinatus* under rice-crayfish farming. The *C. quadricarinatus* were reared at low density (LD), medium density (MD), and high density (HD) for 90 days. The results showed that the superoxide dismutase (SOD), catalase (CAT), glutathione (GSH), and malondialdehyde (MDA) levels were higher in the HD group than those in the LD group. Transcriptomic analysis revealed 1944 upregulated and 1157 downregulated genes in the gills of the HD group compared to the LD group. Gene ontology (GO) enrichment analysis indicated that these differentially expressed genes (DEGs) were significantly associated with ATP metabolism. KEGG (Kyoto Encyclopedia of Genes and Genomes) analysis also showed that high stocking density resulted in the dysregulation of oxidative phosphorylation. Furthermore, high stocking density upregulated six lipid metabolism-related pathways. Overall, our findings, despite the limited number of samples, suggested that high stocking density led to oxidative stress and dysregulation of energy metabolism in the gills of *C. quadricarinatus* under rice–crayfish co-culture. Alteration in energy metabolism may be an adaptive response to adverse farming conditions.

## 1. Introduction

Integrated rice–fish farming is a promising ecological production model that combines rice planting and aquaculture in the same space, with the potential to enhance efficiency and reduce the environmental impact of agriculture [1]. This approach can be used to effectively utilize the unique spatial environment and water resources of rice fields, significantly reducing the use of pesticides and fertilizers [2]. Furthermore, the circular flow of energy and material resources in rice field ecosystems promotes mutual benefits and safe production of both rice and aquatic animals [3]. Notably, in China, integrated rice–fish farming practices have remarkably expanded, covering a vast area of 39.6612 million mu and producing nearly 20 million tons of rice and 3.5569 million tons of aquatic products in 2021 [4]. Several models of integrated rice–fish farming have been developed in China, including rice–carp, rice–turtle, rice–crayfish, rice–crab, rice–loach, and rice–snail [5]. Among these, the rice–crayfish co-culture is the most extensively applied and highest-yielding integrated rice-fish farming model in China [4,6]. Currently, research on rice–crayfish co-culture primarily focuses on its ecological benefits. For instance, rice–crayfish co-culture enhances the utilization rate of carbon sources and the abundance of ammonia-oxidizing bacteria, which is beneficial for improving the soil structure and maintaining the soil nitrogen balance in paddy fields [7]. It also improves the capacity of the soil to sequester carbon and suppress greenhouse gas emissions [8]. Meanwhile, rice-crayfish co-culture has the potential to regulate and enhance biodiversity in rice fields [9].

Stocking density is a fundamental factor that affects aquaculture productivity. High stocking density can facilitate the optimal use of water resources and increase the income of farmers. However, prolonged high-density is a stressor that triggers competition among aquatic species for food and space, ultimately reducing the growth, survival rates, and quality of aquatic animals [10,11,12]. Studies have shown that high stocking densities may lead to negative physiological effects, such as reduced immunity and increased vulnerability to diseases, particularly for crustaceans, which exhibit territorial and aggressive behavior [13]. Research on *Litopenaeus vannamei* has indicated a negative correlation between stocking density, growth performance, and survival rate [14]. Similarly, a high stocking density has been found to decrease the activity of digestive enzymes and inhibit the growth of juvenile *Procambarus clarkia* [15]. In *Marsupenaeus japonicus* juveniles, a positive correlation was observed between the antennal breakage rate and stocking density [16]. Furthermore, a high stocking density has been found to decrease the antioxidant capacity and stress resistance of *L. vannamei* [17]. The core gut microbiota was negatively influenced by high stocking density, which may negatively influence *L. vannamei* performance [18]. However, there is a scarcity of studies available that focus on the physiological and molecular variations of crustaceans under different stocking densities in integrated rice–fish farming systems.

The gills of aquatic animals play a pivotal role in numerous physiological processes, including gas exchange, osmotic/ionic regulation, energy metabolism, ammonia excretion, and detoxification [19]. This organ is highly sensitive to variations in the aquatic environment and is susceptible to the harmful effects of stressors, such as salinity, temperature, and cadmium exposure [20,21,22]. A previous study showed that a high stocking density enhanced carbohydrate, protein, and triglyceride metabolism in the gills of *Eleginops maclovinus* [23]. In *Oncorhynchus mykiss*, high stocking density increases carbonic anhydrase activity and oxygen consumption [24]. Furthermore, high stocking density was found to increase the severity of amoebic gill disease in *Salmo salar* L. [25]. This evidence suggests that it is crucial to maintain suitable stocking densities for the proper functioning of gills in aquatic animals. However, there is currently limited research on the effects of stocking density on gill energy metabolism in crustaceans.

Red claw crayfish (*Cherax quadricarinatus)* is one of the world’s most valuable freshwater shrimps in terms of economics. Since its introduction to China in the 1990s, red claw crayfish has become an important species for aquaculture, primarily for farming in ponds. With the development of integrated rice–fish co-culture, red claw crayfish has become a species for farming in rice fields [26]. In our previous study, we evaluated the effects of stocking density on growth performance and hepatopancreas function in the rice–crayfish co-culture system [27]. However, data on the effects of stocking density on other physiological responses and molecular functions are currently unavailable.

In this study, we aim to assess the effect of stocking density on oxidative stress and energy metabolism in the gills of *C. quadricarinatus* under integrated rice–crayfish farming. To achieve this, we raised crayfish at three different densities for 90 days, and then we examined changes in biochemical parameters and transcriptomes. The findings of this study will provide new insights into the underlying mechanisms of stress induced by high stocking density in *C. quadricarinatus*.

## 2. Results

### 2.1. Changes in Oxidative Stress Parameters

**Figure 1:**
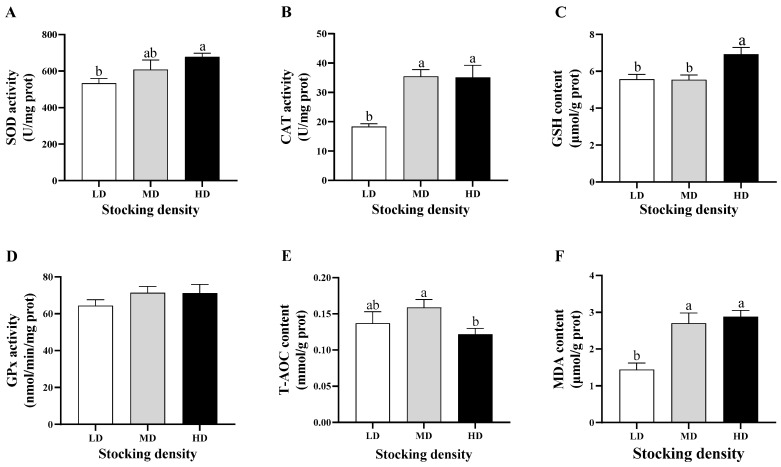
The effects of different stocking densities on oxidative stress parameters in gills of *Cherax quadricarinatus.* (**A**) Superoxide dismutase (SOD). (**B**) Catalase (CAT). (**C**) Reduced glutathione (GSH). (**D**) Glutathione peroxidase (Gpx). (**E**) Total antioxidant capacity (T-AOC). (**F**) Malondialdehyde (MDA). LD, low stocking density; MD, medium stocking density; HD, high stocking density. Different letters a, b denote significant differences (*p* < 0.05).

As shown in Figure 1A–C, the activities of superoxide dismutase (SOD) and catalase (CAT) and reduced glutathione (GSH) content in the gills of the high stocking density (HD) group were significantly higher than those in the low stocking density (LD) group (*p* < 0.05). In addition, the CAT activity in the medium stocking density (MD) group was significantly higher than that in the LD group (*p* < 0.05). However, there was no significant difference in glutathione peroxidase (Gpx) activity in the gills of crayfish among the different density groups (Figure 1D). The total antioxidant capacity (T-AOC) level was increased in the MD group, while it was decreased in the HD and significantly lower than that in the MD group (*p* < 0.05; Figure 1E). Compared to the LD group, malondialdehyde (MDA) content was markedly increased in the MD and HD groups (*p* < 0.05; Figure 1F).

### 2.2. Transcriptome Sequencing and Analysis of Differently Expressed Genes (DEGs) in Gills

RNA-seq data showed that, after filtering, 39,311,670 (99.76%) to 46,816,068 (99.43%) clean reads were obtained, with Q_20_ and Q_30_ values ranging from 97.45% to 97.89% and 92.75% to 93.87%, respectively, and GC content ranging from 37.27% to 41.33% (Table 1). The total mapping ratio ranged from 90.45% to 95.16% (Table 1). These results indicated that the sequencing data of the gills from the LD and HD groups of *C. quadricarinatus* are reliable.

**Figure 2:**
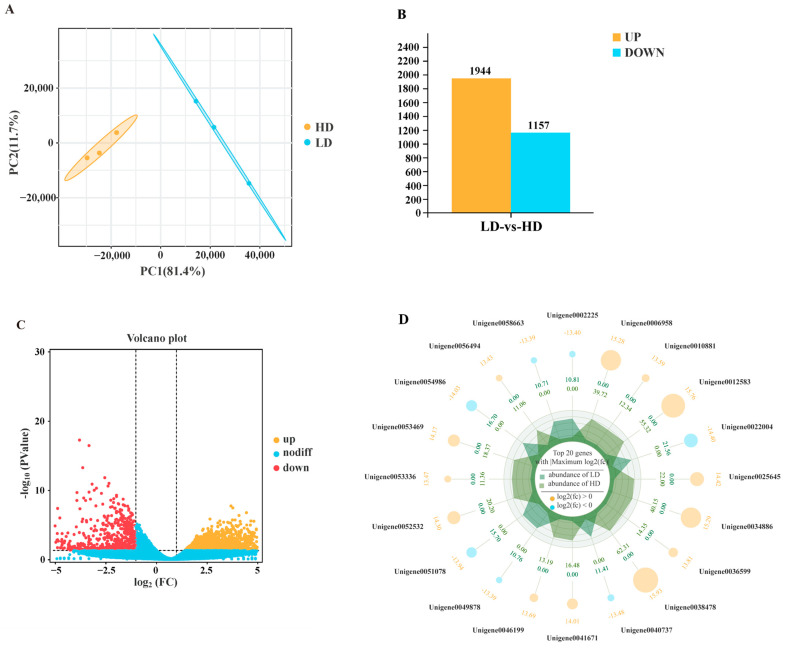
Differently expressed genes (DEGs) in gills of *C. quadricarinatus* between low stocking density (LD) and high stocking density (HD). (**A**) The correlation among samples in LD and HD. (**B**) Significantly upregulated and downregulated genes number in different groups. (**C**) Volcano plot of identified genes in the RNA-seq. (**D**) Top 20 DEGs induced by high stocking density.

The principal component analysis (PCA) result showed that the LD and HD groups were obviously divided into two categories (Figure 2A), indicating that different stocking densities had a significant effect on gene expression patterns in the gill tissue. Analysis of DEGs between the LD and HD groups revealed a total of 3101 genes, with 1944 upregulated genes and 1157 downregulated genes in the gill tissue (Figure 2B,C). Radar plots depicting the data further demonstrated that, among the top 20 DEGs, thirteen genes were upregulated, and seven genes were downregulated in response to changes in stocking density (Figure 2D).

### 2.3. Gene Ontology (GO) Enrichment Analysis of DEGs

**Figure 3:**
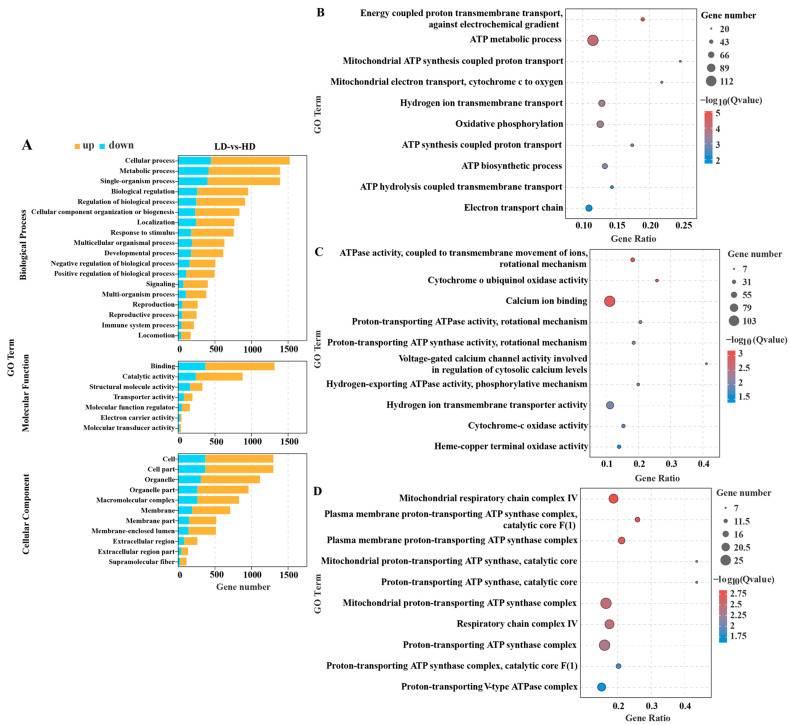
Gene ontology (GO) enrichment analysis for DEGs in gills of *C. quadricarinatus* between low stocking density (LD) and high stocking density (HD). (**A**) GO enrichment for DEGs in biological process, molecular function, and cellular component. (**B**) Significantly enriched GO terms for DEGs in the biological process category. (**C**) Significantly enriched GO terms for DEGs in molecular function category. (**D**) Significantly enriched GO terms for DEGs in the cellular component category.

GO enrichment analysis of DEGs was performed to assess their biological functions (Figure 3A). In the biological process category (Figure 3B), the DEGs were significantly associated with the ATP metabolic process (*p*.adj = 0.00005), ATP biosynthetic process (*p*.adj < 0.001), oxidative phosphorylation (*p*.adj < 0.001), and electron transport chain (*p*.adj = 0.017). In the molecular function category (Figure 3C), the DEGs were enriched in cytochrome-c oxidase activity (*p*.adj = 0.014), cytochrome o ubiquinol oxidase activity (*p*.adj = 0.001), and calcium ion binding (*p*.adj = 0.001). In the cellular component category (Figure 3D), DEGs were primarily enriched in the mitochondrial respiratory chain complex IV (*p*.adj = 0.001), the respiratory chain complex IV (*p*.adj = 0.003), and the proton-transporting ATP synthase complex (*p*.adj = 0.004).

### 2.4. Kyoto Encyclopedia of Genes and Genomes (KEGG) Enrichment Analysis of DEGs

**Figure 4:**
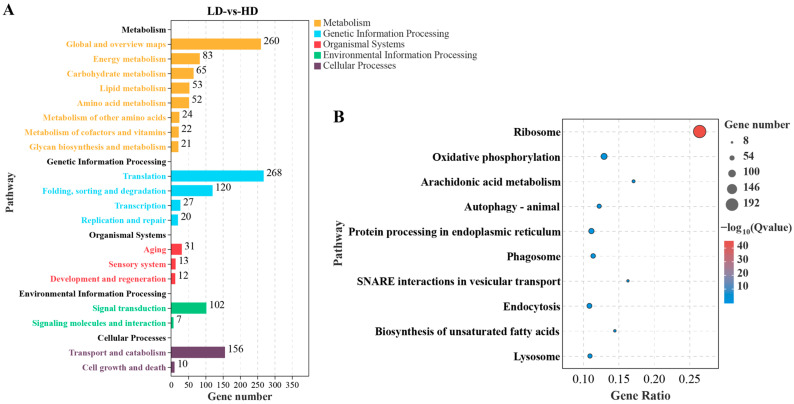
KEGG enrichment analysis for DEGs in gills of *C. quadricarinatus* between low stocking density (LD) and high stocking density (HD). (**A**) DEGs enriched pathways in KEGG A class and B class. (**B**) The top 10 enriched KEGG pathways for DEGs.

The KEGG enrichment analysis showed that 874 DEGs were enriched in 136 pathways of the five KEGG A classes: metabolism, organismal systems, cellular processes, genetic information processing, and environmental information processing (Figure 4A). The top 20 enriched pathways are listed in Figure 4B, with many pathways related to metabolic functions, such as oxidative phosphorylation, arachidonic acid metabolism, and biosynthesis of unsaturated fatty acids. Protein synthesis and processing, including pathways related to ribosomes and protein processing in the endoplasmic reticulum, were also affected to varying degrees by different stocking densities.

### 2.5. DEGs in the Oxidative Phosphorylation and ATP Metabolic Process

**Figure 5:**
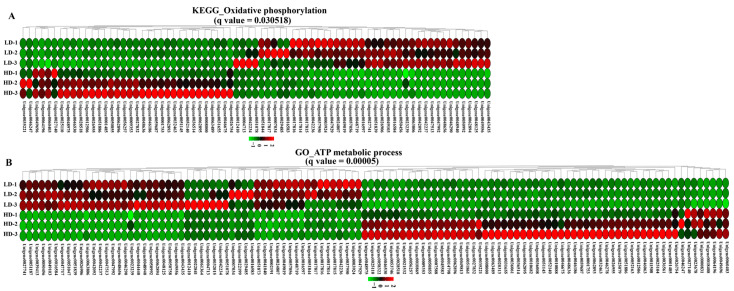
Expression of genes related to oxidative phosphorylation (**A**) and ATP metabolic process (**B**) in the gill of *C. quadricarinatus* between the LD group and the HD group.

After 90 days of farming, oxidative phosphorylation in the gills was considerably altered (*p*.adj = 0.031; Figure 5A). There were 34 upregulated and 41 downregulated genes in the HD group compared with those in the LD group. Specifically, in the NADH dehydrogenase, the gene expressions of the NADH-ubiquinone oxidoreductase chain 1 (ND1, Unigene0047513), ND3 (Unigene0049019), ND4 (Unigene0037950), and ND5 (Unigene0037950) were significantly downregulated in the HD group, while the expressions of NADH dehydrogenase (ubiquinone) Fe-S protein 4 (NDUFS4, Unigene0028518), NDUFS8 (Unigene0009353), NDUFA5 (Unigene0001753), and NDUFA10 (Unigene0036514) were significantly upregulated. In cytochrome c oxidase (COX), the transcriptions of COX2 (Unigene0063806), COX3 (Unigene0048719), COX4 (Unigene0008000), and COX17 (Unigene0051145) were significantly upregulated in the HD group. Similarly, F-type ATPases, including ATPeF1A (Unigene0051886), ATPeF1B (Unigene0062896) and ATPeF1G (Unigene0048636), and V-type ATPase, including ATPeV1B (Unigene0032463), ATP6V1C (Unigene0059687), ATP6V1D (Unigene0056257), and Atp6V1E (Unigene0050855) were upregulated in the in HD group.

The ATP metabolic process was significantly affected by stocking density (*p*.adj = 0.00005; Figure 5B). HD treatment resulted in 112 DEGs, including 58 upregulated and 54 downregulated genes.

### 2.6. Changes in Lipid Metabolism-Related Pathways

**Figure 6:**
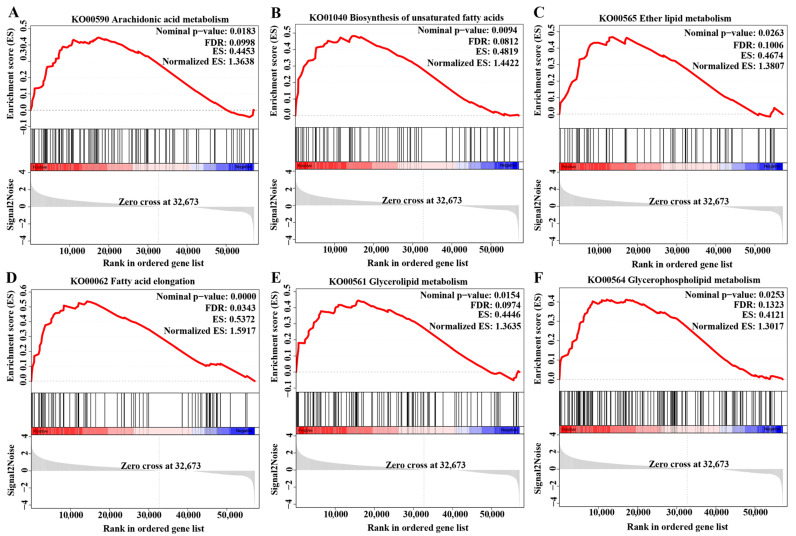
Changes in lipid metabolism-related pathways identified using GSEA in the gill of *C. quadricarinatus* between the LD group and the HD group. (**A**) Arachidonic acid metabolism. (**B**) Biosynthesis of unsaturated fatty acids. (**C**) Ether lipid metabolism. (**D**) Fatty acid elongation. (**E**) Glycerolipid metabolism. (**F**) Glycerophospholipid metabolism. To attain statistical significance, a |Normalized ES| > 1, nominal *p*-value < 0.05, and false discovery rate (FDR) < 0.25 in each gene set were set as threshold values.

Gene set enrichment analysis (GSEA) results showed that stocking density notably impacted lipid metabolism in the gills of *C. quadricarinatus*. In the HD group, six lipid metabolism-related pathways were upregulated, including arachidonic acid metabolism, biosynthesis of unsaturated fatty acids, ether lipid metabolism, fatty acid elongation, glycerolipid metabolism, and glycerophospholipid metabolism (Figure 6).

### 2.7. Validation via RT-qPCR

**Figure 7:**
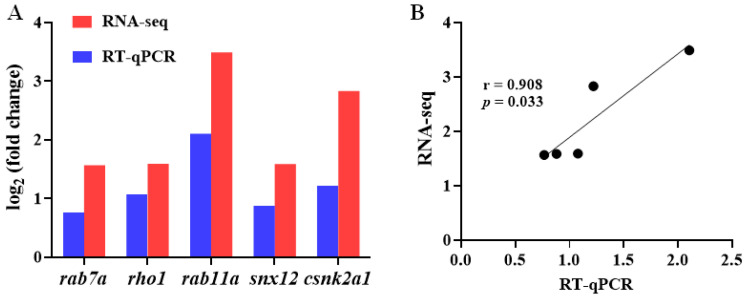
Validation of RNA-seq data via RT-qPCR analysis. (**A**) Comparative analysis of differentially expressed genes by RT-qPCR and RNA-seq. (**B**) The correlation between RT-qPCR data and RNA-seq data.

Five genes were randomly selected to validate the accuracy of the transcriptome results via RT-qPCR analysis. The results showed that the RNA-seq data were significantly consistent with the RT-qPCR data (r = 0.908, *p* = 0.033; Figure 7A), further supporting the reliability of the transcriptome sequencing results.

## 3. Discussion

Stocking density is a key factor in aquaculture that determines production, economic benefits, and resource utilization. Consequently, high stocking densities are common in aquaculture practice. However, high stocking density may cause water quality deterioration, inhibition of aquatic animal growth, and decreased quality. Furthermore, high stocking density as a stressor may activate the stress response system and impair the physiological function and health of farmed aquatic animal [28]. Previous findings have indicated that aquatic animals under high stocking conditions experience oxidative stress, which often manifests as alterations in enzymatic antioxidants (e.g., SOD and CAT) and non-enzymatic antioxidants (e.g., GSH) [29,30]. For example, studies on *O. mykiss* and *S. maxima* have shown that high stocking densities reduced the levels of antioxidant parameters, such as SOD, GSH, and CAT, resulting in impaired antioxidant function [30,31]. Nevertheless, other studies on *L. vannameiin*, *P. olivaceus,* and *Clarias gariepinus* have demonstrated that antioxidants (e.g., SOD, CAT, and GSH) tend to increase with increased stocking density, indicating a positive response of the antioxidant system to stress [32,33,34]. Similarly, we found a significant increase in SOD and CAT activities and GSH content in the gills of *C. quadricarinatus* after being farmed for 90 days, suggesting that *C. quadricarinatus* responded to stress by activating its antioxidant defense system.

Exposure to oxidative stress can lead to excessive amounts of reactive oxygen species (ROS) that attack lipids on the cell membrane and result in lipid peroxidation [35]. MDA is a well known lipid peroxidation product and serves as an indicator of oxidative stress levels [36]. Previous studies have shown that stress induced by high density results in an increased MDA level in various tissues [37]. In this study, the MDA contents in the gills of the MD and HD groups of *C. quadricarinatus* were significantly higher than those in the LD group. These findings are in agreement with previous studies conducted on *O. mykiss* [38] and *Fenneropenaeus chinensis* [39], which also suggested that high density stocking induced lipid peroxidation in the gills of *C. quadricarinatus*.

In general, aquatic animals can trigger physiological responses to adapt and to cope with changes in culture conditions, which require additional energy [40]. ATP plays a critical role as a direct energy source in many cellular process, including cell proliferation, metabolism, and survival [41]. Environmental fluctuations can modify the ATP metabolism in aquatic animals, ultimately affecting their cellular functions and physiological processes [42]. The gills of fish and crustaceans are pivotal organs responsible for gas exchange and osmoregulation, which are limited by the energy supply and are particularly sensitive to environmental changes [43]. Many previous studies have indicated that aquatic animals experience changes in their energy metabolism, owing to environmental stressors. For example, an early study suggested that ATP production in *O. mossambicus* gills was boosted in response to salinity challenges, allowing them to acclimate to adverse environments [44]. In metabolomic analysis, ATP synthase was found to increase in the gills of *Cynoglossus semilaevis* with increasing salinity [45]. Chronic exposure to tributyltin (TBT) decreased ATP levels by altering ATP enzymatic systems in gills of the common carp (*Cyprinus carpio*) [46]. The gills of Matrinxa (*Brycon amazonicus*) exhibit decreased ATP availability, owing to lipid peroxidation and ROS overproduction when exposed to air, leading to a change in their energy supply [47]. In the gills of *Penaeus vannamei*, ammonia stress reduces ATP content and cellular energy levels, which may alter physiological processes in gills [48]. In line with previous findings, in this study, transcriptome analysis revealed that the high stocking density significantly affected ATP metabolic process and ATP biosynthetic process, resulting in the upregulation of 58 genes and the downregulation of 54 genes in the gills of *C. quadricarinatus*. We speculate that the alteration in the ATP metabolic process may be attributed to oxidative stress induced by high stocking density, which could potentially compromise energy supply, gas exchange, and osmoregulation in the gills.

It is a well established fact that the production of cellular ATP is reliant on the process of oxidative phosphorylation that occurs within the mitochondrial electron transport chain [49]. This process is also accompanied by the generation of ROS, which contributes to both homeostatic signaling, as well as oxidative stress during pathological conditions [50]. In crustacean gills, several studies have shown that environmental stress can significantly impact oxidative phosphorylation. For instance, salinity stress alters the oxidative phosphorylation pathway in the gills of *Eriocheir sinensis*, leading to changes in energy–metabolism homeostasis [51]. Similarly, *L. vannamei* responded to nitrite stress by upregulating genes related to oxidative phosphorylation, resulting in enhanced energy metabolism in gills [52]. Chronic hypoxic stress affects the oxidative phosphorylation pathway in the gills of *Macrobrachium nipponense*, potentially coping with increased energy demand [53]. In our study, KEGG analysis demonstrated that oxidative phosphorylation was the most enriched pathway in the gills, with 34 upregulated and 41 downregulated genes. Meanwhile, cytochrome c oxidase, F-type ATPase, and V-type ATPase in the oxidative phosphorylation pathways were significantly upregulated in the HD group. These changes may represent an adaptation to supply more energy to cope with a crowded environment. Moreover, this may explain the alterations in ATP metabolic processes and oxidative stress under high stocking density.

Lipid metabolism plays a crucial role in the energy supply of organisms because fatty acids serve as a significant source of ATP [54]. In aquatic animals, this process is highly susceptible to environmental stress, particularly at high stocking density. An early study found that *Sparus aurata* mobilized its triglyceride content to fulfill the increased energy demand under high rearing density [55]. Similarly, *O. niloticus* adapts to high density conditions by altering fatty acid composition and stimulating lipid metabolic enzymes [56]. Transcriptomic analysis of *Ctenopharyngodon idella* also revealed that lipid metabolism is accelerated at high stocking density [57]. However, conflicting findings have been reported in the literature. For example, high stocking density in *Brachymystax lenok* led to the activation of energy metabolism while repressing lipid metabolism [58]. Similarly, in *Micropterus salmoides*, high stocking density tends to suppress fatty acid metabolism, biosynthesis of unsaturated fatty acids, and steroid biosynthesis [59]. It is worth noting that our data were consistent with former studies, as we found that six lipid metabolism-related pathways were upregulated in the gills of *C. quadricarinatus* under high stocking density conditions. These findings suggest that enhanced lipid metabolism may provide a coping mechanism to counteract stress caused by high stocking density.

To minimize experimental errors caused by individual variability, a pooled sample was employed for transcriptome sequencing in this study, which was a common approach in studies of aquatic animals, such as *Chlamys farreri* [60,61], *Rachycentron canadum* [62], *Danio rerio* [63], *Plectropomus leopardus* [64], *E. sinensis* [65], and *Oryzias melastigma* [66]. Despite the limited sample size of three (each sample contains fifteen individuals) in the study, it satisfied the requirements for transcriptome analysis, and numerous published reports have demonstrated that three samples are acceptable for transcriptome analysis [67,68,69,70,71,72,73,74,75]. Furthermore, the primary objective of this study was to investigate the impact of stocking density on *C. quadricarinatus* populations, and, therefore, individual effects were disregarded.

## 4. Materials and Methods

### 4.1. Crayfish, Experimental Design and Sampling

**Figure 8:**
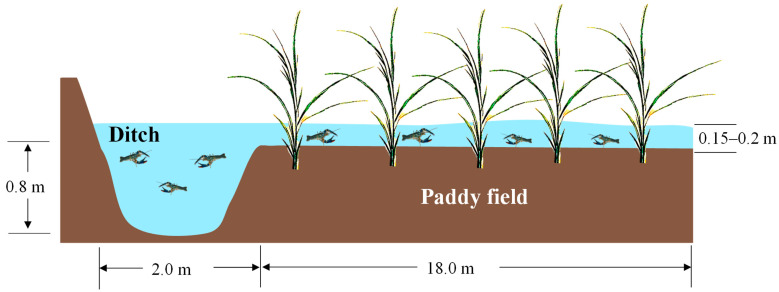
Schematic diagram of the integrated rice–crayfish farming system in this study.

The experiment was conducted at the Jingjiang Farming Base of the Freshwater Fisheries Research Center (Jingjiang, China). The red-clawed crayfish with a 14.29 ± 1.05 g average initial weight was provided by the Zhejiang Freshwater Fisheries Research Institute (Huzhou, China). Nine rice–crayfish co-culture systems were used (Figure 8) in this experiment. The area of each rice–crayfish co-culture system was 400 m^2^, including a 360 m^2^ rice field and a 40 m^2^ canal refuge (ditch, 0.8 m in depth). In each co-culture system, the rice variety (Nangeng 5055) was planted in each rice field.

In this experiment, the crayfish were randomly assigned to three groups: low stocking density (LD), medium stocking density (MD), and high stocking density (HD). Each group consisted of 3000, 6000, and 9000 crayfish, respectively, distributed among the three replicates. Throughout the 90-day experimental period, crayfish were fed once a day with a commercial feed containing 30% crude protein, 3% crude fat, 8% crude fiber, 18% crude ash, 1% total phosphorus, and 1 to 3.5% calcium. The paddy field was managed following conventional local agricultural practices.

At the end of the experiment, 45 individuals from each group were randomly selected, and the gills were sampled under anesthesia on ice. For the analysis of oxidative stress parameters, the gills of five individuals were pooled into a single sample, whereas the gills of 15 individuals were combined into a sample for transcriptome sequencing. All samples were temporarily stored in liquid nitrogen and transferred to long-term storage at −80 °C. It is important to note that the use of crayfish in this study was approved by the Freshwater Fisheries Research Centre (Wuxi, China), and all experimental procedures were performed with proper regard for animal welfare requirements.

### 4.2. Measurement of Oxidative Stress Parameters

The frozen gill samples were thawed on an ice plate, and approximately 0.1g of each sample was homogenized in normal saline (0.86%). The homogenate was centrifuged at 3600 r/min and 4 °C for 10 min, and the supernatant was collected to determine the oxidative stress parameters. Oxidative stress parameters, including T-AOC, SOD, GSH, Gpx, CAT, and MDA, were measured using commercial kits. The T-AOC level was measured using the Ferric Reducing Ability of Plasma (FRAP) method [76], while SOD activity was tested via the water-soluble tetrazolium-1 (WST-1) method [77]. GSH and MDA contents were determined using the 5,5′-dithiobis-(2-nitrobenzoic acid) (DTNB) method [78,79] and the thiobarbituric acid (TBA) method, respectively. CAT activity was measured by analyzing the decomposition of H_2_O_2_ [80], and total protein in the gills was measured using the bicinchoninic acid (BCA) method [81].

### 4.3. Transcriptome Sequencing and Analysis

Total RNA was isolated from the gill tissue using a commercial kit (Trizol reagent, Invitrogen, Carlsbad, CA, USA), following the manufacturer’s protocol. After assessing the quality of the RNA, we enriched the mRNA by removing the rRNA. The enriched mRNA was fragmented into short fragments and reverse-transcribed into first-strand cDNA. Second-strand cDNA was synthesized using DNA polymerase I, RNase H, dNTP, and buffer. The cDNA was subjected to end-repair, A-base addition, adapter ligation, and PCR amplification to obtain the final library. The library was subsequently sequenced using an Illumina Novaseq 6000 by Gene Denovo Biotechnology Co. (Guangzhou, China), and the raw data were deposited in the open database (Sequence Read Archive database, No. PRJNA970695).

Raw reads from the sequencing machines were filtered using Fastp (version 0.18.0) [82] to obtain high-quality clean reads. The clean reads were utilized for subsequent assembly using the Trinity software (v2.1.1) and evaluated for integrity using BUSCO [83]. Annotations were assigned to the resulting unigenes using established databases, including Nr, KEGG, KOG, and SwissProt. The expression of unigenes was calculated and normalized to RPKM (Reads Per kb per Million reads).

Based on the gene expression in each sample, principal component analysis (PCA) was conducted to reveal correlations among the samples. The differentially expressed genes (DEGs) between LD and HD were analyzed using DESeq2 software (version 3.0) with a significance threshold *p* < 0.05 and |log_2_ Fold Change (FC)| > 1 [84]. To gain insight into the functions of the DEGs, we performed functional enrichment analysis, referring to GO and KEGG databases. GO and KEGG enrichment analysis were performed using Goseq software (version 2.12) and KOBAS 2.0 software, respectively, and significantly enriched GO terms and KEGG pathways were defined by a hypergeometric test with a significance threshold *q* value < 0.05 (corrected by the Benjamin–Hochberg method). Furthermore, we employed gene set enrichment analysis (GSEA) to identify specific KEGG pathways between the LD and HD groups, and |Normalized enrichment score (ES)| > 1,Nominal *p*-value < 0.05, and FDR < 0.25 in each gene set were set as threshold values. All analyses and transcriptome charts generated using the OmicShare tools (www.omicshare.com, accessed on 1–23 April 2023)

### 4.4. Quantitative Real-Time PCR (RT-qPCR) Analysis

To validate the accuracy of the transcriptome sequencing, we performed RT-qPCR analysis. Total RNA was extracted from gill tissue using RNAiso Plus reagent (Takara, Beijing, China), along with chloroform and isopropyl alcohol (Sinopharm Chemical Reagent Co., Ltd., Shanghai, China). The RNA concentration was measured using a Nanodrop 2000 spectrophotometer (Thermo Scientific, Waltham, MA, USA), and quality was evaluated using the Agilent 4200 system (Agilent Technologies, Santa Clara, CA, USA) with RNA integrity number (RIN) > 7. After quality assessment, RNA was reverse-transcribed into cDNA using the primeScript™ RT reagent kit (Takara). In brief, 1 μg of RNA was mixed into gDNA Eraser (Takara) to remove the genomic DNA at 42 °C for 2 min. After that, the reaction mixture was supplemented with 1 μL of PrimeScript RT Enzyme Mix I, 1 μL of RT Primer Mix, 4 μL of 5× PrimeScript Buffer, and 4 μL of RNase Free dH_2_O. The mixture was then incubated at 37 °C for 15 min and 85 °C for 5 s.

The cDNA was then amplified using a commercial kit (Takara, RR820A) to determine the Cq value of the target gene. Briefly, the reaction mixture for RT-qPCR (total volume 25 μL) included 12.5 μL of TB Green Premix Ex Taq II (Takara), 2 μL of cDNA, 1 μL of forward and reverse specific primers each, and 8.5 μL of Rnase-free water. The RT-qPCR reaction was carried out under the following conditions: initial denaturation at 95 °C for 30 s, followed by 40 cycles of denaturation at 95 °C for 5 s, and extension at 60 °C for 1 min. After 40 cycles, the specificity of the PCR product was confirmed by analyzing the melting curve. Relative expression of the gene was calculated using the 2^−∆∆Cq^ method, with β-actin serving as the reference gene [85]. The specific primers for the target genes analysis in this study can be found in Table 2.

### 4.5. Statistical Analysis

Data in the study were analyzed using SPSS (version 25.0) and EXCL software (version 2016). All results are expressed as mean ± SE (standard error). The normal distribution of the data was analyzed using the Shapiro–Wilk test, and the variance homogeneity of the data was analyzed using the Levene test. Differences in oxidative stress parameters among different groups were analyzed using one-way analysis of variance (ANOVA), followed by the least significant difference (LSD) test. The correlation between RT–qPCR and RNA-seq was assessed using the Pearson test. Statistical significance was established when the *p*-value was less than 0.05.

## 5. Conclusions

This study identified, for the first time, changes in energy metabolism in the gills of crustaceans under high stocking density. High stocking density induced oxidative stress and lipid peroxidation in the gills of *C. quadricarinatus*. Further, we conducted transcriptome analysis to examine the molecular mechanisms underlying the stress caused by high stocking density in the gills. After 90 days of farming, we identified 3101 DEGs in the gill tissue between the LD and HD groups, with 1944 upregulated genes and 1157 downregulated genes. GO and KEGG enrichment revealed that the DEGs significantly enriched ATP metabolic process and oxidative phosphorylation pathway. Meanwhile, high stocking density caused the upregulation trend in six lipid metabolism-related pathways. The changes in ATP metabolic process, oxidative phosphorylation, and lipid metabolism may be an adaptive response to stress induced by high stocking density. Our study contributed to understanding the mechanism that *C. quadricarinatus* responded to the adverse conditions in the rice–crayfish farming system.

## Figures and Tables

**Table 1:** Statistical information of the sequencing data.

Samples	Raw Reads	Clean Reads	Q_20_ (%)	Q_30_ (%)	GC (%)	Total Mapping Ratio (%)
LD-1	42,902,116	42,656,312 (99.43%)	97.56%	93.09%	41.17%	90.45%
LD-2	47,083,210	46,816,068 (99.43%)	97.89%	93.87%	41.33%	91.42%
LD-3	42,388,836	41,955,622 (98.98%)	97.45%	92.75%	40.67%	93.92%
HD-1	42,661,940	42,553,024 (99.74%)	97.73%	93.28%	38.01%	94.14%
HD-2	43,082,230	42,968,088 (99.74%)	97.69%	93.18%	37.27%	95.16%
HD-3	39,407,166	39,311,670 (99.76%)	97.84%	93.50%	37.44%	94.97%

Note: Q_20_ and Q_30_, the base quality scores, were no less than 20 and 30, respectively, for clean reads. GC, GC content of clean reads.

**Table 2:** Specific primer sequences for RT-qPCR in the study.

Gene	Primer Sequence (5′-3′)	Efficiency (%)
ras-related protein Rab-7A (*rab7a*)	F: TTTGGGATACAGCCGGTCAA R: CTCGCTTTGTTGATACCGCC	98.7
ras-like GTP-binding protein (*rho1*)	F: TGATAGACTGCGACCCCTCT R: ACTGGCTCTTGTTTCATCTTCTGA	99.2
ras-related protein Rab-11A-like protein (*rab11a*)	F: GTCAGCAGTAGGGAGAGAGC R: TTGTTGGAGGAAGATCCGCTA	98.6
sorting nexin-12 (*snx12*)	F: GCCTCGTCTCCAAGAAACAA R: AACCACAATCTTGCTGTCCCT	99.1
casein kinase II subunit alpha (*csnk2a1*)	F: ATAGATTGGGGGTTGGCGGA R: TGAAGTATCTGGAGGCCACTCT	98.9
*β-actin*	F: ATCACTGCTCTGGCTCCTGCTACC R: CGGACTCGTCGTACTCCTCCTTGG	98.7

## Data Availability

The raw data of transcriptome sequencing are available at Sequence Read Archive (SRA) data-base of NCBI (No. PRJNA970695).
